# A Conceptual Analysis of Food Parenting Practices in the Light of Self-Determination Theory: Relatedness-Enhancing, Competence-Enhancing and Autonomy-Enhancing Food Parenting Practices

**DOI:** 10.3389/fpsyg.2018.02373

**Published:** 2018-11-29

**Authors:** Roberta Di Pasquale, Andrea Rivolta

**Affiliations:** ^1^Department of Management, Information and Production Engineering, University of Bergamo, Bergamo, Italy; ^2^Child Protection Service Sondrio, Sondrio, Italy

**Keywords:** child obesity, food parenting practices, self-determination theory, parenting dimensions, positive parental involvement, provision of structure, autonomy support

## Abstract

The relationships between parenting behaviors and child obesity or obesogenic eating behaviors (i.e., overeating unhealthy food) have been studied through the use of different parenting constructs; in particular, specific food parenting practices have been extensively investigated, but there is currently a need for a more comprehensive and integrative theoretical framework to guide future investigations. The present article argues for the use of Self-determination theory as a valuable framework to conceptually organize food parenting practices relevant to children’s obesogenic eating behaviors. The three parenting dimensions of positive parental involvement, autonomy support and provision of structure - highlighted by Self-determination theory as closely linked to the process of internalization of healthy behaviors and values by the child – will be adopted as a framework to categorize food parenting practices into three types of practices: relatedness-enhancing, competence-enhancing and autonomy-enhancing.

## Introduction

Childhood obesity, is regarded as an extremely serious health problem, resulting from a complex and dynamic pattern of ineffective regulation of eating behavior combined with a lack of physical activity and prolonged sedentary periods, rooted in a specific physical and social obesogenic environment (Birch and Anzman, 2010; Kaushal and Rhodes, 2014). Moreover, the condition of overweight and obesity, is seen to generate negative psychosocial consequences such as peer stigmatization (Di Pasquale and Celsi, 2017) and victimization (Gray et al., 2009) which are likely to reinforce dysfunctional eating patterns in children.

The notion that parents – as the primary socializing agents of a child’s eating style – can play an important role in either contrasting or unintentionally encouraging obesogenic eating behaviors – and ultimately childhood obesity – seems to be theoretically uncontroversial and empirically well supported (Ventura and Birch, 2008). The association between child obesity-related eating behaviors and parenting has been investigated through the use of different parenting constructs. General parenting style (Baumrind, 1971), with particular reference to the Maccoby and Martin (1983), typology has been used as a theoretical framework to explain the relationship between parenting and child overweight/obesity or child obesity-related behaviors (Collins et al., 2014). Also, the notion of domain-specific feeding styles has been proposed as a conceptual tool to better understand the influence of parenting on child obesogenic eating behaviors (Wardle et al., 2002; Hughes et al., 2005). Finally, a host of specific food parenting practices – namely the concrete, goal-directed behaviors adopted by parents to directly influence their children’s eating behavior – have been extensively investigated. However, research results (Vollmer and Mobley, 2013; Shloim et al., 2015) indicate that the relationship between child overweight or obesity and single parenting constructs is often mixed or inconsistent, especially in the case of specific food parenting practices, partially due to a lack of conceptual clarity in defining constructs (Vaughn et al., 2013).

For this reason, in the last few years different contributions have been devoted to the creation of a more articulated conceptual framework to organize food parenting practices related to child obesogenic eating behaviors in order to develop a common conceptual vocabulary and guide future investigations. In the present article we will argue that a particularly useful theoretical framework can be found in Self-determination theory (Ryan and Deci, 2000), a general theory of motivation that offers a specific dimensional model of parenting related to children’s internalization of parental norms and values.

## Food Parenting Practices: Contemporary Classifications and Conceptual Models

In the last few years there have been a number of attempts to systematize constructs in the field of food parenting practices, by classifying and clustering different practices into functionally homogeneous categories, and by building comprehensive models based on a limited number of higher-order fundamental constructs.

Gevers et al. (2014) have conducted a Delphi study with the aim of classifying food parenting practices constructs, with a specific focus on the consumption of snacks. They have grouped several descriptive statements representing concrete instances of food parenting practices into 18 conceptual categories, and then used a 3-factor model of higher-order parenting constructs to further classify the different food parenting practices categories. The first construct, named *Control*, refers to directive, restrictive and punitive parenting practices aimed at prompting the child to meet parental demands regarding snack and general food consumption. According to the authors, Control comprises several food parenting practices: *pressure to eat*, namely, pressuring the child to consume healthy food, or to finish healthy food even when not hungry; *emotional feeding*, namely, the use of snacks to comfort the child or to otherwise respond to the child’s emotional distress; *instrumental feeding*, namely using snacks to convince the child to do something, *monitoring* the child’s eating by keeping track of the number and kind of snacks consumed, *rules* setting around snack consumption, and, finally, *permissiveness* regarding the child’s prerogative to consume snacks as they wish. The second construct, named *Responsiveness*, refers to the use of a warm and autonomy-supportive parental attitude aimed at encouraging the child’s self-assertion and individuality in the feeding domain. Responsiveness is thought to comprise food parenting practices such as *involving* the child in the purchase, selection and preparation of food; *discussing* food choices and preferences with the child; *educating* the child about the importance of healthy food consumption; *encouraging* the child to eat a large variety of healthy foods; direct *modeling* of healthy eating styles by consuming healthy food and avoiding snacks when the child is present; *rewarding* the child for healthy eating; and *providing feedback* when the child indulges in eating snacks. The third construct, named *Structure*, refers to parental efforts to organize the physical and social feeding environment in order to promote the child’s competence. Structure is understood as comprising food parenting practices such as granting high *accessibility* of healthy food to the child; ensuring high *availability* and *visibility* of healthy food at home, setting *meal routines* such as eating together as a family, and providing a fixed temporal *structure* for snack time.

In another recent contribution, Vaughn et al. (2016), proposed a particularly articulated model of food parenting practices. With this aim, the authors grouped several food parenting practices into three general constructs defined along the lines of general literature on parenting. The first construct, named *Coercive control*, has been characterized in terms of parental efforts to dominate, pressure or impose their will upon the child in the feeding domain. Coercive control encompasses food parenting practices like *food restriction*, namely the parent-centered, authoritarian limitation of the amount and kind of palatable food that the child is allowed to access and consume; *pressure to eat*, namely parental insistence that the child consumes more (healthy) food; *threats and bribes*, namely parental attempts to influence the child’s eating behavior by using either desired food or desired objects/activities as incentives; and finally, *using food to control negative emotions*, that is using food to calm the child when he or she is upset, fussy, bored and so on. The second construct, named *Structure*, has been characterized as a different, non-coercive kind of parental control, focused on increasing children’s competence in adopting desired behaviors in the feeding domain. Structure is seen to comprise several food parenting practices such as setting clear and consistent *rules and limits* regarding food consumption; offering *limited or guided choice* among adequate food options, *monitoring* the child’s food consumption; creating consistent and pleasurable *meal and snack routines*; *modeling* healthy eating in front of the child; ensuring differential *availability* and *accessibility* of healthy and unhealthy food at home; adopting techniques and strategies for *food preparation* to provide healthy food alternatives; and finally, using *unstructured practices*, namely lack of parental control deriving from a neglectful or overly indulgent attitude in the feeding domain. The third construct, named *Autonomy promotion/support*, has been characterized in terms of fostering children’s independence and autonomous self-regulation in the feeding domain, by developing a sense of ownership of parental-endorsed norms. Autonomy promotion includes food parenting practices like *nutrition education*, namely parental efforts to transmit information and skills regarding healthy eating in order to enable the child to make informed choices; children’s *involvement* in meal planning and preparation, gentle *encouragement* to adopt healthy habits, *praise* as a means of positive reinforcement for the child’s healthy eating behavior; *reasoning* as a means to convince the child to adopt healthy eating behaviors; and, finally, *negotiation* as a means to resolve differences between parents and the child around food consumption.

Both Gevers and colleagues’ contribution and that of Vaughn and colleagues appear to draw theirs higher-order constructs from the general literature on parenting, including parenting constructs related to Self-determination theory, as will be discussed further in this paper. In particular, the proposed model incorporates a fundamental feature of contemporary parenting models, the bipartition of parental control into a negative, coercive form and a more positive, regulatory form (Grolnick and Pomerantz, 2009). However, the two models appear to almost completely neglect the parenting dimension that has been variously named warmth, acceptance, support, involvement, which represents the most fundamental construct in the general literature on parenting. (Rollins and Thomas, 1979; Rohner, 1986; Barber et al., 2005).

## Conceptualizing Food Parenting Practices Through the Framework of Self-Determination Theory

Most studies investigating how parenting influences behavior related to child obesity are based on the basic – yet often implicit – conception that in order to engage in behavior linked to healthy eating and refrain from adopting unhealthy eating – children need to be motivated by their parents through the use of specific parenting practices. Thus, considering the implicit relevance of motivational processes, Self-determination theory (Ryan and Deci, 2000) can prove to be an appropriate framework for conceptualizing the role played by different food parenting practices in motivating children to adopt healthy eating habits.

Self-determination theory (Deci and Ryan, 2000; Ryan and Deci, 2000; is in fact regarded as a general motivation theory that centers on the dichotomy between self-determined, volitional behaviors and those that are externally coerced or internally pressured. The theory also focuses on the distinction between behaviors guided by intrinsic motivation, and behaviors guided by extrinsic motivation. Intrinsically motivated behavior refers to what a person does on his or her own because of the interest, pleasure or satisfaction derived from that particular behavior. Conversely, extrinsically motivated behavior is enacted because it is perceived to be instrumental to an independent consequence. Behavior that is generally enacted as a way to conform to social norms (i.e., healthy eating patterns and physical activity) is considered to be extrinsically motivated. This is not to say that the person necessarily perceives to be coerced or pressured into taking on this behavior: following the Self-determination framework, such behavior can become self-determined through the process of internalization.

Internalization is a proactive process and consists in a progressive transformation from behavior regulated by external contingencies (i.e., praise, criticism, material rewards and punishments, etc.) to behavior regulated by internal processes (inherent interest in an activity, alignment with internal values). When externally regulated behaviors are perceived to help function effectively in the social world, the individual is inherently motivated to integrate these behaviors into the self, thus promoting the process of internalization.

Moreover, Self-determination theory suggests that the process of internalization is facilitated by the fulfillment of three psychological needs: *relatedness*, generally defined in terms of the experience of interpersonal contact, warmth and affection; *competence*, generally defined in terms of the degree of mastery one has within their environment; and *autonomy*, generally defined in terms of being the origin of one’s behavior.

Using the Self-determination framework to look at the way parenting influences children’s obesity-related eating behaviors thus implies highlighting the role food parenting practices play in promoting or undermining the child’s internalization of healthy norms and behavior around eating. Self-determination theory provides a detailed model through which we can understand the process of behavioral self-regulation and internalization of social norms. In addition, through the Self-determination framework, the parenting behaviors that promote or hinder this process of internalization can be identified.

## Parenting Dimensions at Play in the Child’s Process of Internalization

A tripartite dimensional model of parenting was developed following the conceptual lines of Self-determination theory (Grolnick and Ryan, 1989; Grolnick et al., 1997), and is based on three parenting dimensions: *parental involvement, provision of structure* and *autonomy support.* These dimensions are held to be highly connected to the three basic psychological needs of autonomy, competence and relatedness, which in turn are seen to foster the child’s autonomous integration and internalization of social norms and values.

The construct of *positive parental involvement* includes parental warmth and affection, and can be better understood as the degree of the parent’s attention and dedication toward the child and his or her optimal development (Grolnick and Slowiaczek, 1994). Positive involvement can be seen in parents who are interested in and aware of what goes on in their child’s life and play an active role. Through their behavior, parents who are involved in a positive way provide emotional and concrete resources that foster the child’s confidence and sense of self-direction. Positive involvement also satisfies the child’s need for relatedness, and is thus believed to reinforce the child’s identification and autonomous internalization of the social requests and values promoted by the parents. It should be noted that in addition to a lack of involvement, there are also inadequate forms of parental involvement (Pomerantz and Eaton, 2001; Pomerantz et al., 2005, 2007) that are thought to have a negative impact on the process of internalization of parental norms.

*Provision of structure* refers to the parent’s ability to provide clear and consistent guidelines, expectations, and rules regarding the child’s behavior along with clear feedback and consistent consequences. This approach is believed to foster the child’s sense of being competent in taking on socially desired norms and behaviors. With regards to the school/academic domain, Farkas and Grolnick (2010) identified several elements characteristic of an adequate provision of structure: clear and consistent rules, guidelines, and expectations regarding the child’s academic life; realistic parental expectations; predictable consequences; feedback about the child’s performance; explanation of parental rules and requests; and finally, willingness on the parent’s part to enforce rules by exerting an adequate level of parental authority.

Finally, *Autonomy support* refers to the transmission of social norms and demands from parent to child in a way that takes into account the child’s point of view and involves him or her in decision-making processes and independent problem solving. It is believed that a parenting style characterized by autonomy support strengthens the child’s autonomy, thus promoting the internalization of behaviors and values. Conversely, in the *coercive* parenting style the parent considers only his or her own perspective and attempts to motivate the child’s behavior through threats and rewards, punitive disciplinary strategies, and/or psychological pressure exerted by inducing guilt or withdrawing love. This coercive style negatively impacts the degree to which the child experiences him or herself as an autonomous agent, and consequently hinders the autonomous internalization of parental norms and values.

A final point to underline is that in Self-determination theory autonomy support and provision of structure are conceived as two virtually independent parenting dimensions. Therefore, parents can provide a child with an adequate structure in an autonomy supportive way as well as in a coercive and controlling way. On the other hand, parental involvement seems to be, at least partially, a precondition of both autonomy support and provision of adequate structure. Autonomy supportive parenting behaviors (such as acknowledging the child’s perspective, offering options and negotiating) are in fact likely to require a greater amount of psychological and material resources, as well as more time, than coercive strategies, such as threats, bribes and psychological pressure. Similarly, the resources needed for an adequate provision of structure are greater than those used in unstructured parenting behavior, which is characterized by a lack of consistent rules, monitoring and follow-through.

## Positive Parental Involvement and Relatedness-Enhancing Food Parenting Practices

From a Self-determination theory perspective, parental positive involvement, both in general parenting and in the specific feeding domain, can be regarded as the most important precondition for promoting an effective (that is, autonomous) internalization of healthy eating behaviors and values by children. In fact, children’s sense of relatedness and positive connection to their parents, built on shared positive experiences in the feeding domain, can act as a powerful facilitating condition for the transmission of healthy eating habits, This is due to the fact that children are more willing to accept parents’ socializing efforts and internalize parent-endorsed behaviors, norms and values in the feeding domain when they experience a sense of relatedness to their parents in their socializing functions.

The parenting dimension of positive involvement appears to be almost totally neglected in studies investigating parental influences on child obesity-related eating behaviors. Furthermore, there is insufficient specific consideration for either parental positive involvement or any equivalent construct (parental warmth, parental support) in recent contributions aimed at categorizing food parenting practices to construct comprehensive models.

Parenting practices such as asking the child to help preparing food or involving the child in the purchase and selection of healthy foods (*child involvement* in Gevers and colleagues’ classification), as well as eating meals together as a family (a component of *mealtime routines* in Vaughn and colleagues’ model) can be primarily regarded as *relatedness-enhancing food parenting practices;* this refers to practices through which parents can facilitate the child’s autonomous self-regulation and internalization of norms regarding healthy feeding styles by communicating their positive involvement, interest and care for the child in the feeding domain, and by enhancing the child’s sense of relatedness.

Moreover, some of the parenting practices that contribute to promoting healthy child eating behavior and which are usually located in the parenting dimension of structure – such as high availability of a wide variety of healthy foods, could at least in part also be considered relatedness-enhancing practices. The aforementioned practices, in fact, are clearly suitable to convey to the child the parent’s interest and commitment, thus fostering the child’s sense of relatedness.

Conversely, absent or inadequate parental involvement in the feeding domain can undermine parent’s socializing efforts around healthy eating. Indeed, inadequate involvement can take different forms; in the first place, it can be characterized in terms of an absence of the aforementioned positive, relatedness-enhancing food parenting practices: no common meals, no shared selection and purchase of food, etc. In the second place, even specific negative parenting practices such as parents’ use of unhealthy food (i.e., sweets) as bribes to induce good behavior or as a means to regulate children’s negative emotions, could be interpreted, such as the previous point, as a form of lacking or inadequate involvement, and be defined as *relatedness-thwarting food parenting practices*, since they represent an attempt to compensate for the absence of emotional support and coaching for the child, that, in turn, the child may experience in terms of a diminished sense of relatedness to the parents.

## Parental Provision of Structure and Competence-Enhancing Food Parenting Practices

From a Self-determination theory perspective, parental provision of structure in the feeding domain is crucial to promote children’s sense of competence in conforming to parental norms regarding food consumption, in achieving an adequate capacity to self-regulate eating behaviors, and ultimately in engaging in healthy eating habits. Children need to be put in the condition to feel competent in self-regulating their eating behavior according to parental norms and requests, and in adopting a healthy eating style: in this respect, the organization of the food environment by parents plays an important role.

Food parenting practices such as providing clear and consistent rules about the kind and quantity of foods that the child is allowed to eat, and offering a sufficient availability and accessibility of healthy versus unhealthy food can be regarded as *competence-enhancing food parenting practices*, since they represent a prototypical instance of adequate provision of structure suitable for enabling the child to competently meet parental requests around healthy eating; moreover, feeding practices such as nutrition education, namely the use explicit didactic techniques to encourage consumption of healthy food, or the direct modeling of healthy eating behavior by the parents should also be categorized in this parenting dimension.

In the literature on food parenting practices, parental provision of structure in the feeding domain has been increasingly recognized as a central construct, and clearly distinct from more coercive forms of control. Nevertheless, even in recent conceptual maps and models, the concept of structure has been presented as an almost homogeneous, unipolar construct. In the light of Self-determination theory, it seems more useful to view parental structure as a bipolar construct, in particular by contrasting adequate forms of structure with inadequate forms of structure. characterized by lacking or ineffective practices through which parents attempt to promote children’s competence in adopting a healthy eating style. In this respect, the absence of consistent rules about food consumption, the modeling of unhealthy eating behavior, and the high availability and accessibility of unhealthy food in the house can be seen as *competence-thwarting food parenting practices*, since they represent instances of inadequate parental structure that make it more difficult for the child to conform to parental norms and requests regarding healthy eating.

## Parental Autonomy Support and Autonomy-Enhancing Food Parenting Practices

The final parenting dimension highlighted by Self-determination theory as essential in promoting children’s effective self-regulation and autonomous, integrated motivation is autonomy support. In the feeding domain, this parenting dimension can be interpreted in terms of engaging the child in volitionally enacting not intrinsically motivated healthy eating behaviors (i.e., consumption of healthy food, such as fruits, vegetables and other nutritious foods), as well as in committing the child to avoid unhealthy, eating (such as palatable energy-dense food overeating).

A number of food parenting practices associated with a child’s healthy eating behavior, such as discussing and negotiating with the child food choices and preferences, or allowing the child a guided choice among several healthy foods, can be interpreted specifically as *autonomy-enhancing food parenting practices*; in fact, through these practices parents can better promote the child’s autonomous internalization of healthy norms regarding eating behavior, specifically by fostering his/her sense of autonomy and personal ownership of parent-endorsed norms and values.

The models and taxonomies of food parenting practices illustrated above, acknowledge autonomy support/promotion as a fundamental higher-order construct, and directly refer to Self-determination theory as a relevant conceptual source. Nonetheless, in the light of Self-determination theory some inconsistencies can be identified in the way in which some food parenting practices – such as parental praise, encouragement and nutrition education – have been assigned to this higher-order construct.

According to a Self-determination theory perspective, autonomy-supportive parenting in the feeding domain can be regarded as opposite from a coercive or controlling parenting, in which parental efforts to socialize the child around healthy eating are based on external coercion or internalized forms of psychological pressure. Similarly, autonomy-enhancing food parenting practices can be contrasted with *autonomy-thwarting food parenting practices*, such as the use of physical and psychological forms of parental pressure on the child to eat healthy food as well as the coercive restriction of unhealthy food. In this respect, the models illustrated in the first part of the article are consistent with the conceptual underpinning of the construct of coercive control as defined by Self-determination theory. Conversely, constructs like emotional feeding or using food to control negative emotions should be assigned to the negative pole of the parental involvement dimension and regarded mainly as relatedness-thwarting food parenting practices.

## Conclusion

Self-determination theory appears to be a useful theoretical framework for conceptually organizing food parenting practices in the context of child obesogenic eating behaviors, given its specific focus on children’s internalization of parental norms and values. The three general parenting dimensions emphasized in Self-determination theory, namely parental involvement, provision of structure and autonomy support have been connected to parenting behaviors in the feeding domain. This has resulted in a tripartite categorization of food parenting practices according to the different ways in which they contribute to the child’s internalization of healthy eating norms and behaviors. Specifically, *relatedness-enhancing* food parenting practices pursue the goal of internalization by strengthening the child’s sense of relatedness to the parents as socializing agents in the feeding domain; *competence-enhancing* food parenting practices do so by fostering the child’s ability to conform to a healthy eating style; finally, *autonomy-enhancing* food parenting practices pursue said goal by promoting the child’s sense of autonomy in the feeding domain.

## Author Contributions

All authors contributed equally to the conception and the writing of the article.

## Conflict of Interest Statement

The authors declare that the research was conducted in the absence of any commercial or financial relationships that could be construed as a potential conflict of interest.

## References

[B1] Barber B. K. Stolz H. E. Olsen J. A. (2005). Parental support, psychological control, and behavioral control: assessing relevance across time, culture, and method. *Monogr. Soc. Res. Child Dev.* 70 1–137. 16359423 10.1111/j.1540-5834.2005.00365.x

[B2] Baumrind D. (1971). Current patterns of parental authority. *Dev. Psychol. Monogr.* 4 1–103. 10.1037/h0030372

[B3] Birch L. L. Anzman S. L. (2010). Learning to eat in an obesogenic environment: a developmental systems perspective on childhood obesity. *Child Dev. Perspect.* 4 138–143. 10.1111/j.1750-8606.2010.00132.x

[B4] Collins C. Duncanson K. Burrows T. (2014). A systematic review investigating associations between parenting style and child feeding behaviours. *J. Hum. Nutr. Diet.* 27 557–568. 10.1111/jhn.12192 24386994

[B5] Deci E. L. Ryan R. M. (2000). The “what” and “why” of goal pursuits: human needs the self-determination of behavior. *Psychol. Inq.* 11 319–338. 10.1207/S15327965PLI1104_01 27055568

[B6] Di Pasquale R. Celsi L. (2017). Stigmatization of overweight and obese peers among children. *Front. Psychol.* 8:524. 10.3389/fpsyg.2017.00524 28473781 PMC5397522

[B7] Farkas M. S. Grolnick W. S. (2010). Examining the components and concomitants of parental structure in the academic domain. *Motiv. Emo.* 34 266–279. 10.1007/s11031-010-9176-7

[B8] Gevers D. W. M. Kremers S. P. J. de Vries N. K. van Assema P. (2014). Clarifying concepts of food parenting practices. a delphi study with an application to snacking behavior. *Appetite* 79 51–57. 10.1016/j.appet.2014.04.002 24732407

[B9] Gray W. N. Kahhan N. A. Janicke D. M. (2009). Peer victimization and pediatric obesity: a review of the literature. *Psychol. Sch.* 46 720–727. 10.1002/pits.20410 15678228

[B10] Grolnick W. S. Deci E. L. Ryan R. M. (1997). “Internalization within the family: the self-determination theory perspective,” in *Parenting and Children’s Internalization of Values: A Handbook of Contemporary Theory*, eds Grusec J. E. Kuczynski L. (Hoboken, NJ: John Wiley), 135–161.

[B11] Grolnick W. S. Pomerantz E. M. (2009). Issues and challenges in studying parental control: toward a new conceptualization. *Child Dev. Perspect.* 3 165–170. 10.1111/j.1750-8606.2009.00099.x 20161628 PMC2813058

[B12] Grolnick W. S. Ryan R. M. (1989). Parent styles associated with children’s self-regulation and competence in school. *J. Educ. Psychol.* 81 143–154. 10.1037/0022-0663.81.2.143

[B13] Grolnick W. S. Slowiaczek M. (1994). Parents’ involvement in children’s schooling: a multidimensional conceptualization and motivational model. *Child Dev.* 65 237–252. 10.1111/j.1467-8624.1994.tb00747.x 8131650

[B14] Hughes S. O. Power T. G. Fisher J. O. Mueller S. Nicklas T. A. (2005). Revisiting a neglected construct: parenting styles in a child-feeding context. *Appetite* 44 83–92. 10.1016/j.appet.2004.08.007 15604035

[B15] Kaushal N. Rhodes R. E. (2014). The home physical environment and its relationship with physical activity and sedentary behavior: a systematic review. *Prev. Med.* 67 221–237. 10.1016/j.ypmed.2014.07.026 25084562

[B16] Maccoby E. E. Martin J. (1983). “Socialization in context of the family: parent-child interaction,” in *Handbook of Child Psychology* Vol. 4 ed. Hetherington E. M. Newark, NJ: Science and Education Publishing.

[B17] Pomerantz E. M. Eaton M. M. (2001). Maternal intrusive support in the academic context: transactional socialization processes. *Dev. Psychol.* 37 174–186. 10.1037/0012-1649.37.2.174 11269386

[B18] Pomerantz E. M. Moorman E. A. Litwack S. D. (2007). The how, whom, and why of parents’ involvement in children’s academic lives: more is not always better. *Rev. Educ. Res.* 77 373–410. 10.3102/003465430305567

[B19] Pomerantz E. M. Wang Q. Ng F. F. (2005). Mothers’ affect in the homework context: the importance of staying positive. *Dev. Psychol.* 41 414–427. 10.1037/0012-1649.41.2.414 15769196

[B20] Rohner R. P. (1986). *The Warmth Dimension: Foundations of Parental Acceptance–Rejection Theory*. Beverly Hills, CA: Sage.

[B21] Rollins B. C. Thomas D. L. (1979). “Parental support, power, and control techniques in the socialization of children,” in *Contemporary Theories About the Family: Research-Based Theories*, eds Burre W. R. Hill R. Nye F. I. (New York, NY: Free Press), 317–364.

[B22] Ryan R. M. Deci E. L. (2000). Self-determination theory and the facilitation of intrinsic motivation, social development, and well-being. *Am. Psychol.* 55 68–78. 10.1037/0003-066X.55.1.68 11392867

[B23] Shloim N. Edelson L. R. Martin N. Hetherington M. M. (2015). Parenting styles, feeding styles, feeding practices, and weight status in 4–12-year-old children: a systematic review of the literature. *Front. Psychol.* 6:1849. 10.3389/fpsyg.2015.01849 26696920 PMC4677105

[B24] Vaughn A. Tabak R. Bryant M. Ward D. (2013). Measuring parent food practices: a systematic review of existing measures and examination of instruments. *Int. J. Behav. Nutr. Phys. Act.* 10:61. 10.1186/1479-5868-10-61 23688157 PMC3681578

[B25] Vaughn A. Ward D. Fisher J. Faith M. S. Hughes S. O. Kremers S. P. (2016). Fundamental constructs in food parenting practices: a content map to guide future research. *Nutr. Rev.* 74 98–117. 10.1093/nutrit/nuv061 26724487 PMC4892304

[B26] Ventura A. K. Birch L. L. (2008). Does parenting affect children’s eating and weight status? *Int. J. Behav. Nutr. Phys. Act.* 5:15. 10.1186/1479-5868-5-15 18346282 PMC2276506

[B27] Vollmer R. L. Mobley A. R. (2013). Parenting styles, feeding styles, and their influence on child obesogenic behaviors and body weight. a review. *Appetite* 71 232–241. 10.1016/j.appet.2013.08.015 24001395

[B28] Wardle J. Sanderson S. Guthrie C. A. Rapoport L. Plomin R. (2002). Parental feeding style and the inter-generational transmission of obesity risk. *Obes. Res.* 10 453–462. 10.1038/oby.2002.63 12055321

